# Interplay between Histone and DNA Methylation Seen through Comparative Methylomes in Rare Mendelian Disorders

**DOI:** 10.3390/ijms22073735

**Published:** 2021-04-03

**Authors:** Guillaume Velasco, Damien Ulveling, Sophie Rondeau, Pauline Marzin, Motoko Unoki, Valérie Cormier-Daire, Claire Francastel

**Affiliations:** 1 Université de Paris, Epigenetics and Cell Fate, CNRS UMR7216, 75013 Paris, France; guillaume.velasco@univ-paris-diderot.fr (G.V.); damien.ulveling@scipio.bio (D.U.); 2 Imagine Institute, Université de Paris, Clinical Genetics, INSERM UMR 1163, Necker Enfants Malades Hospital, 75015 Paris, France; sophie.rondeau@inserm.fr (S.R.); pauline.marzin@inserm.fr (P.M.); valerie.cormier-daire@inserm.fr (V.C.-D.); 3 Division of Epigenomics and Development, Medical Institute of Bioregulation, Kyushu University, Fukuoka 812-8582, Japan; unokim@bioreg.kyushu-u.ac.jp

**Keywords:** rare diseases, DNA methylation profiling, histone methylation, biomarkers, epigenetic crosstalks

## Abstract

DNA methylation (DNAme) profiling is used to establish specific biomarkers to improve the diagnosis of patients with inherited neurodevelopmental disorders and to guide mutation screening. In the specific case of mendelian disorders of the epigenetic machinery, it also provides the basis to infer mechanistic aspects with regard to DNAme determinants and interplay between histone and DNAme that apply to humans. Here, we present comparative methylomes from patients with mutations in the de novo DNA methyltransferases DNMT3A and DNMT3B, in their catalytic domain or their N-terminal parts involved in reading histone methylation, or in histone H3 lysine (K) methylases NSD1 or SETD2 (H3 K36) or KMT2D/MLL2 (H3 K4). We provide disease-specific DNAme signatures and document the distinct consequences of mutations in enzymes with very similar or intertwined functions, including at repeated sequences and imprinted loci. We found that KMT2D and SETD2 germline mutations have little impact on DNAme profiles. In contrast, the overlapping DNAme alterations downstream of NSD1 or DNMT3 mutations underlines functional links, more specifically between NSD1 and DNMT3B at heterochromatin regions or DNMT3A at regulatory elements. Together, these data indicate certain discrepancy with the mechanisms described in animal models or the existence of redundant or complementary functions unforeseen in humans.

## 1. Introduction

Cytosine methylation is a relatively stable component of mammalian epigenomes that collectively define cell identity and are essential for normal development [1]. In mammals, the covalent transfer of a methyl group onto DNA occurs mainly in a CpG context. DNA methylation (DNAme) has been assigned major roles in fundamental biological processes that include the regulation of gene expression programs and the silencing of inactive-X chromosome in females, imprinted genes, transposons, and repetitive elements [2,3,4]. DNAme is catalyzed by DNA methyltransferases (DNMTs) among which DNMT3A and DNMT3B add methyl groups de novo to unmethylated DNA, whereas DNMT1 maintains DNAme patterns from hemi-methylated DNA after DNA replication has occurred [5,6]. The information provided by DNAme is conveyed by “readers,” i.e., factors that recognize and bind to methylated sites, which in turn recruit various effectors of chromatin remodeling complexes to promote transcriptional activation or repression depending on genomic and cellular contexts [7,8].

An important question in the field is to understand how the DNAme machinery is guided to specific methylation sites. Several mechanisms, not mutually exclusive, have been proposed, including the recruitment by transcription factors to specific sequences [9,10], which lent meaning to the coincidence between the most variable methylated sites of the human genome and the density of transcription factor binding sites (TFBS) [11]. In addition, an important crosstalk between methylation of histone tails and that of DNA has come to the forefront. At genomic regions with hallmarks of repressive heterochromatin, which include DNAme, trimethylation of histone H3 on lysine 9 or 27 (H3K9me3 or H3K27me3) or histone H4 on lysine 20 (H4K20), a complex reciprocal relationship between the corresponding histone methyltransferases (KMTs) and the DNAme machinery was shown to participate in the specification of places where DNAme is established and maintained [12]. Moreover, the discovery that DNMTs have the ability to recognize certain histone marks through discrete domains in their N-terminal part has shed new light on the tight interplay between histone modifications and DNAme. In addition to their C-terminal catalytic domain, the de novo DNMT3 enzymes contain a proline–tryptophan–tryptophan–proline (PWWP) and an ATRX-DNMT3A-DNMT3L-type zinc finger (ADD) domain [13]. The PWWP domain was shown to recognize lysine 36 trimethylation of histone H3 (H3K36me3) enriched at transcribed gene bodies and active enhancers in mice [10,14]. The ADD domain of DNMT3 proteins can recognize and bind unmodified lysine 4 of histone H3 (H3K4me0), whereas the interaction is disrupted by any larger modification of H3K4 [15,16]. Lysine methylation is catalyzed by SET (Su(var)3-9, Enhancer-of-zeste and Trithorax) domain-containing KMTs and removed by Jumonji (Jmj) domain-containing histone demethylases (KDM). Among these, we can cite SETD2/KMT3A, thought to be the only KMT involved in the tri-methylation of H3K36 in humans, whereas other proteins of the KMT3 family (also known as Nuclear Receptor Binding SET Domain Proteins NSD1–3) catalyze mono- or di-methylation [14,17]. Similar to the other histone modifications, H3K36 methylation is reversible and can be actively removed by KDMs of the KDM2/JHDM1 and KDM4/JHDM3/JMJD2 with a JmjC signature domain [18]. The dynamic transition between H3K4 and its methylated forms is mediated by SET-domain containing KMTs of the KMT2 family (also known as Myeloid/Lymphoid Or Mixed-Lineage Leukemia; MLL) and H3K4 histone demethylases of the KDM5/JARID1C family of enzymes. Hence, the typical mark of active CpG-Island (CGI) promoters, H3K4me3, functions as a DNAme blocker that would explain why these promoters are mostly unmethylated [19]. In a more general way, it is clear that methylation of histone H3 on lysines K4, K9 and K36 plays important roles in the action of de novo DNMTs in mammalian genomes, although understanding the genomic and developmental stage specificities of these crosstalks remains to be fully elucidated, at least in the context of human cells.

DNAme patterns are highly dynamic in germ cells and during pre-implantation embryonic development where they participate in the regulation of tissue-specific gene expression and allow for differential cell fate determination during lineage specification [20,21,22,23]. Post-implantation, DNAme and its machinery are also required for the correct differentiation capacity of adult stem cells in a number of tissues, such as the hematopoietic or nervous systems, to reprogram transcriptional outputs and instruct lineage specification [24,25,26]. Not surprisingly, DNAme and its machinery are essential for the healthy development of mammals and, more generally, throughout life. Evidence comes from animal models [4,5,27], but also from human diseases where DNAme landscapes or the DNAme machinery itself are often impaired, such as in cancer or other complex multifactorial disease [28,29]. In addition, the expanding class of genetic diseases caused by rare pathogenic variants of the epigenetic machinery has led to a real breakthrough in the field in allowing to infer direct connections between epigenetic actors, multi-loci alterations and the emergence of disease phenotypes [30,31,32]. Given the interplay between histone and DNAme methylation, the question of their coordination depending on the stage of development or differentiation and the genomic loci considered is a current issue which is difficult to answer in humans. In that respect, the identification of germline pathogenic variants in KMTs or KDMs will undoubtedly provide insights into the importance of the histone and DNA methylation crosstalk in setting up DNAme landscapes during development, and into the in vivo contribution of the non-catalytic domains of DNMTs.

Here, we focused on rare early onset developmental disorders with loss-of-function (LoF) pathogenic variations in DNMT3A (Tatton-Brown-Rahman syndrome; TBRS) [33] and in DNMT3B (Immunodeficiency Centromeric instability and Facial dysmorphism; ICF) [34]. We compared their DNAme patterns with that of patients with mutations in KMTs, as well as with patients who have mutations in the PWWP or ADD domains of the DNMT3 enzymes. In addition to identifying DNAme signatures with diagnostic value, this comparative methylome opens new avenues for our understanding the functional crosstalks between DNA and histones methylation that applies to humans.

## 2. Results

### 2.1. Non-Redundant Functions of De Novo DNMTs or H3K36 KMTs Are Reflected in Distinct DNAme Alterations Downstream of Their Mutations

In the context where DNAme is established by de novo DNMT3 enzymes, which themselves act as readers of histone methylation in complex regulatory networks, we aimed at profiling DNAme alterations in a set of rare developmental diseases caused by LoF mutations in de novo DNMT3 enzymes, or in H3K36 or H3K4 KMTs (Supplementary Figure S1A). We used published or newly established array-based methylomes [Illumina Human Methylation 450K (HM450K) or EPIC BeadChip arrays; see Material and Methods] from patients with pathogenic variations in *DNMT3A* in Tatton-Brown Rahman (TBRS) [33], *DNMT3B* in Immunodeficiency Centromeric instability and Facial anomalies (ICF1) [34], *NSD1/KMT3A* in Sotos [35], *SETD2* in Sotos-like [36] [also known as Luscan Lumish [37], and *KMT2D/MLL2* in Kabuki [38] syndromes. Of note, and for a comparison with already established DNA methylomes in whole blood from ICF1 [39] and TBRS [40] patients with mutations in catalytic domains of DNMT3B and DNMT3A, respectively, we established new DNAme profiles in patients with non-catalytic (nCD) mutations in these enzymes (Supplementary Figure S2), for whom DNAme profiling had never been established. We also included patients with genome-wide DNAme alterations caused by mutations in non-DNMT factors, i.e., in transcription factors ZBTB24 and CDCA7 (ICF2 and ICF3) or in the chromatin remodeler HELLS (ICF4), for which little overlap of DNAme alterations with that of ICF1 patients had already been documented [39]. Finally, we included two ICFX patients, i.e., ICF patients without known genetic cause, that we had classified as ICF1 based on hypomethylation of pericentromeric Satellite repeats but absence of hypomethylation at centromeric alpha-satellite repeats [41,42], and an hypomethylation signature at germline gene promoters [43]. Therefore, we will refer to ICF1-X patients from now on.

**Figure 1:**
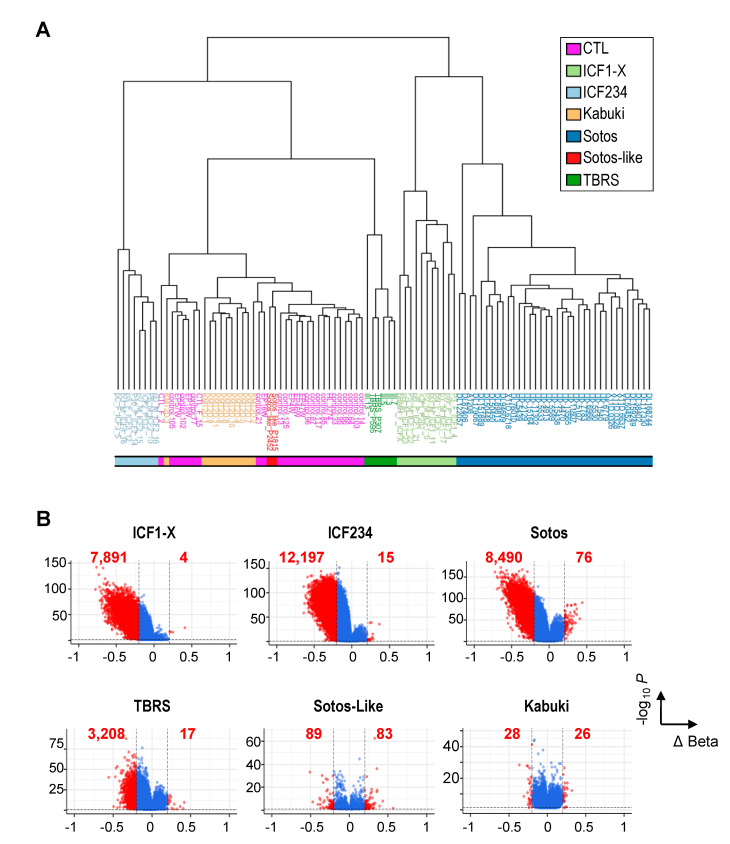
Genome-wide DNA methylation profiling discriminates inherited disorders of the epigenetic machinery. (**A**) Unsupervised clustering of non-affected individuals (CTL) and patients based on the total Differentially Methylated Probes (DMP) identified (28,102 probes). The colored bar at the bottom indicates groups of patients according to their diagnosis. (**B**) Volcano plots of DMPs between patients and non-affected individuals. The mean differences in β-values (ΔBeta) are plotted on the X axis and their statistical significance [−log10 (*p*-value)] on the Y axis. DMPs with a ΔBeta ≥ 0.2 and *p*-values ≤ 0.05, further considered for analysis, are plotted as red dots. The number of hypo- (ΔBeta ≤ −0.2) and hyper- (ΔBeta ≥ 0.2) methylated probes are indicated in red for each group of patients.

We first sought to provide a comprehensive set of differentially methylated probes (DMP) between patients and a set of age- and gender-matched healthy individuals (CTL), focusing on the most reliable ones with a difference in DNAme values Δβ ≥ 0.2 and a *p* value ≤ 0.05 (Supplementary Table S1). As already reported, the set of DMPs identified was sufficient to cluster individuals according to their genotype (Figure 1A and Supplementary Figure S1B). We verified that the potential confounding effect of age of the patients did not impact the clustering of patients according to their genotype and clinical signs (not shown and [39]).

Notable exceptions to the above were the Kabuki and Sotos-like patients, who clustered with the CTL group although they remained distinguishable one from another. Indeed, Kabuki or Sotos-like patients exhibited very little alterations of DNAme landscapes using these higher-than-usual criteria (Figure 1B), with a somewhat remarkable equal repartition between hypo- and hyper-methylated probes (HypoMP and HyperMP). Only 54 probes showed differential methylation as a consequence of mutations in a H3K4 KMT in Kabuki patients with Δβ ≥ 0.2. Surprisingly, mutations in SETD2, the supposedly non-redundant KMT for H3K36me3, also caused very little changes in DNAme patterns in Sotos-like patients (172 DMPs). Hence, it is not surprising that these two groups of patients are not segregated from CTL subjects.

**Figure 2:**
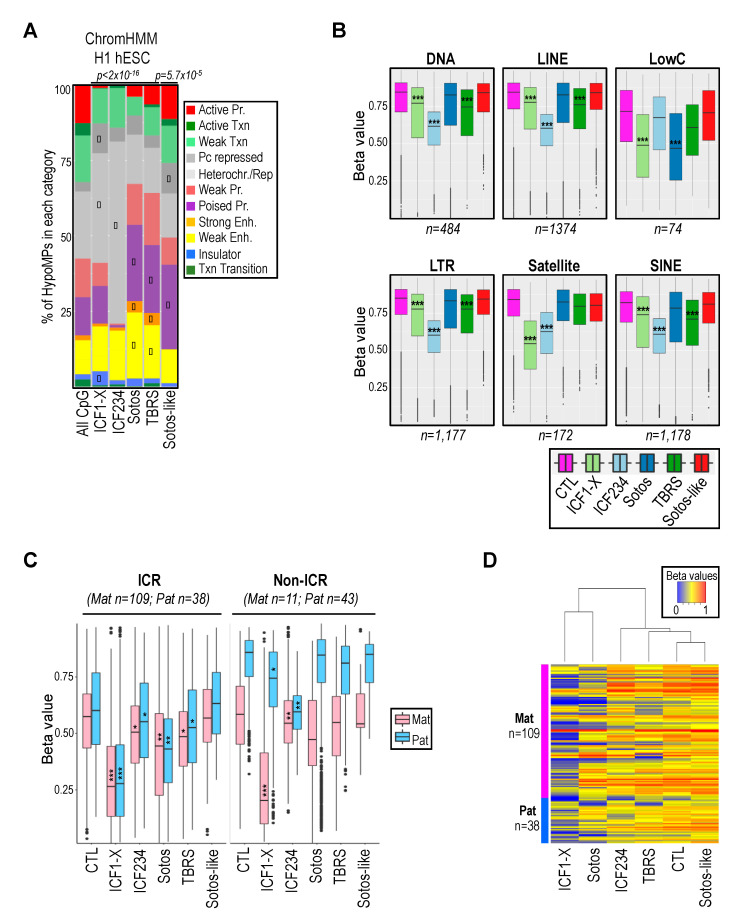
Functional annotation of HypoMPs and distribution across imprinted loci and classes of DNA repeats. (**A**) Distribution of HypoMPs for each group of patients relative to chromatin states established in H1 hESCs (ChromHMM track from ENCODE/Broad Institute). All CpG: repartition of the total number of the HM450K probes analyzed (*n* = 361,359). *p*-values (*p*, Chi-square test) assessing significant changes in the distribution of HypoMPs within the different categories relative to HM450K array composition. The diamond symbol denotes categories that contribute the most to the *p*-values based on the standardized residuals. (**B**,**C**) Distribution of mean β-values for HM450K probes designed in classes of (**B**) DNA repeats or (**C**) imprinted loci (ICR and non ICR), for each group of subjects. For each class, the corresponding number of HypoMPs among all identified DMPs (28,102) is indicated into brackets. Each class of repeats, or maternal (Mat; pink) and paternal (Pat; blue) imprinted loci, are indicated on the top of each box plot. *p*-values (*p*, Wilcoxon test) assessing significant changes in β-values relative to CTL are indicated as ***: *p* < 2 × 10^−14^; **: *p* < 2 × 10^−10^; *: *p* < 2 × 10^−3^. (**D**) Clustering analysis with heat map of mean β-values of the total HypoMPs identified in maternal (Mat; *n* = 109) and paternal (Pat; *n* = 38) ICRs. Each column represents a group of subjects and each row represents a probe. High methylation levels are shown in red and low methylation levels in blue, according to the scale bar above the figure.

In clear contrast, significant alterations to DNAme landscapes were observed in patients with mutations in de novo DNMT3 enzymes (7895 DMPs in ICF1 and 3225 DMPs in TBRS), but also with mutations in a H3K36me2 KMT in Sotos patients (8566 DMPs) (Figure 1B). The vast majority of DMPs were hypomethylated and remarkably comparable in number between ICF1 and Sotos patients, while 2 to 3 times fewer probes were affected in TBRS (3225 DMPs). When addressing the CpG content and position of HypoMPs relative to gene features, we confirmed the remarkable enrichment of HypoMPs in CpG-poor regions (“open sea”) and intergenic regions in ICF2–4 cells (Supplementary Figure S3). HypoMPs in the other diseases did not show striking enrichment in a specific category, with the exception of Sotos-like. Although they have far fewer HypoMPs, more than half of them were found in CpG-rich regions (CGI and shore) and gene bodies. We also interrogated the chromatin states of HypoMPs compared to that established in pluripotent human embryonic stem cells (hESCs; male H1 cell line), a stage at which most of the DNAme landscapes are established [44,45]. This comparison confirmed that the loci with reduced DNAme in ICF cells were largely enriched in genomic regions classified as “heterochromatin” or “repeat-containing loci,” regardless of their genetic origin (Figure 2A). In contrast, the repartition of HypoMPs into functional genomic elements in Sotos and TBRS patients showed significant enrichment in regulatory elements, such as “poised promoter,” “weak enhancer” and “strong enhancer” (Figure 2A). Surprisingly, SETD2 LoF mostly affected the “Polycomb-repressed” category linked to H3K27me3 (Figure 2A).

Gene Ontology (GO) analysis of genes linked to HypoMPs in ICF patients showed enrichment for Biological Processes associated with cell-cell adhesion, ion transport and synapse organization, as previously described, although functional pathways related to sensory perception and skin development was a specific feature of ICF2 to 4 patients, mainly due to the reported DNAme alterations at genes with monoallelic expression that include Olfactory Receptors (OR) and Keratin Associated Protein/Late Cornified Envelope (KRTAP/LCE) genes [39]. In contrast, HypoMPs identified in Sotos and TBRS patients were linked to genes related to embryonic development and morphogenesis, consistent with their growth defects (Supplementary Figure S4 and Table S2), whereas too few HypoMPs in Sotos-like patients prevented their categorization in Biological Processes.

In sum, DNAme profiling was sufficient to segregate patients according to their genotype and identified (epi)genomic signatures of altered DNAme patterns downstream of NSD1 or DNMT3A mutations that were qualitatively more similar to one another than to those caused by DNMT3B LoF, consistent with their partially overlapping spectra of phenotypes.

### 2.2. Hypomethylation of Satellite Repeats and Parental Imprints Are Hallmarks of ICF Patients but Not Patients with Overgrowth Syndromes

Since DNAme is markedly enriched at repetitive DNA sequences [2], which are in turn often affected in human diseases [46], we interrogated the methylation status of the 78,268 probes of the HM450K array that are designed in repetitive elements. These repeats were broken down into six families: Long Interspersed Nuclear Elements (LINE), Short Interspersed Nuclear Elements (SINE), Long Terminal Repeat elements (LTR), Low Complexity repeats (LowC), DNA repeat elements (DNA) and Satellite repeats (Satellite). Consistent with previous studies showing an enrichment of HypoMPs in the “repetitive DNA” and “Heterochromatin” category ([39,47,48] and Figure 2A), all repeat categories exhibited a remarkable reduction of DNAme downstream of ICF mutations, especially in non-DNMT factors (Figure 2B and Supplementary Figure S5). In addition, hypomethylation of satellite repeats was a hallmark of all ICF patients, as it was not observed in other diseases of the epigenetic machinery, at least the ones included in this study (Supplementary Figure S6). In contrast, most DNA repeats remained mostly unaffected by other pathogenic variations, although a small but significant decrease in DNAme at DNA, LINE, LTR, SINE or LowC repeats was respectively observed in TBRS and Sotos patients. LowC repeats are GC-rich sequences that overlap with CGI promoters of coding and non-coding genes and correspond in part to the poised promoters identified using chromatin segmentation of HypoMPs (Figure 2A).

A symptomatic feature of all these syndromes is the abnormal growth of patients, with overgrowth in Sotos, TBRS and Sotos-like or growth delay in ICF patients, associated with intellectual disability (ID) [30,49]. Hence, we first focused on HM450K probes designed in imprinted loci [50,51] at which epigenetic alterations affect their specific dosage and lead to abnormalities of fetal growth and brain functions [52]. Surprisingly, DNAme at parental imprints was dramatically affected by DNMT3B mutations in ICF1 patients (Figure 2C,D and Supplementary Tables S1–S6, columns “Imprinted gene” and “ICR”). More specifically, maternal imprints were affected by DNMT3B mutations and, to a much lesser extent, by NSD1 and DNMT3A mutations, both outside of Imprinted Control Regions (ICR) and at the ICRs themselves (Figure 2C,D). In contrast, paternal imprints outside of the ICRs were mainly affected by mutations in non-DNMT factors in ICF2–4 patients and mostly represented by the Prader-Willi locus on chromosome 15q11-13, as already reported [39].

These results were suggestive of a quite unexpected potential crosstalk between DNMT3B and NSD1-mediated H3K36me2 for the methylated status of certain maternal imprints (Supplementary Tables S1 and S3).

### 2.3. Hypomethylation of Developmentally Regulated Genes Is Shared between ICF, TBRS and Sotos Patients

Interestingly, GO analysis (Supplementary Figure S4 and Tables S7–S10) also identified HypoMPs associated with families of genes encoding transcriptional regulators that are essential for normal embryonic development and fidelity of patterning expressed only at certain stages of development or individual body segments, such as Homeobox (HOX), Chromobox (CBX), Forkhead Box (FOX), SRY-Box (SOX) and Paired Box (PAX) genes. DNAme at these genes was globally more affected in ICF1-X, Sotos and TBRS patients, who also shared HypoMPs throughout the HOX gene clusters (Supplementary Figures S7 and S8).

These data were suggestive of collaborative pathways implicating the corresponding enzymes NSD1, DNMT3A or DNMT3B for CpG methylation at developmentally regulated genes.

### 2.4. DNMT3 and NSD1 Mutations Have Overlapping Consequences for DNAme Alterations in Patients

To identify distinguishing and unifying DNAme signatures in patients included in the study, we then compared the sets of HypoMPs identified in each disease (Supplementary Figure S9A). As expected from the classification of HypoMPs according to (epi)genomic features exposed above, we first found a poor overlap between DNAme landscapes in TBRS and ICF1-X patients (only 35 shared HypoMPs), consistent with DNMT3 enzymes having distinct genomic preferences [53]. We already reported the striking distinguishing methylomes between ICF1 and ICF2–4 patients (827 uniquely shared probes out of 7891 and 12,197 HypoMPs, respectively), which we had interpreted as ZBTB24, CDCA7 and HELLS not being mere platforms for the initial recruitment of DNMT3B at DNAme sites [39]. Likewise, comparing methylomes of ICF2–4 to that of TBRS patients with DNMT3A mutations, we found even fewer shared probes (61 shared probes) (Supplementary Figure S9A), questioning again the functional links between the non-DNMT ICF factors and the DNAme machinery during early development.

In fact, we found the greatest overlap of HypoMe probes between patients with mutations in NSD1 or in one of the two de novo DNMTs, consistent with the known link between H3K36 methylation and recruitment of DNMT3 enzymes at methylation sites (1636 and 1085 unique probes shared between NSD1 and DNMT3B or DNMT3A mutants, respectively) (Supplementary Figure S9A). However, the intersection of methylomes of patients with mutations in NSD1, DNMT3A or DNMT3B showed very poor overlap (63 shared HypoMPs) consistent with the distinct preferences of DNMT3A or B for di- or tri-methylation of H3K36, respectively [10,54,55]. Of note, the few HypoMPs in patients with SETD2 mutations also showed poor overlap with HypoMPs found in ICF1 (15 unique probes) or TBRS (9 unique probes) patients. Together, these data suggested that the disruption of initial establishment of H3K36 di- (NSD1) or tri-methylation (SETD2) patterns have distinct consequences for DNAme patterns.

In sum, this comparative analysis highlighted a functional interplay between writers of H3K36me2, but not H3K36me3, and DNMT3 enzymes in shaping DNAme landscapes in humans.

### 2.5. Catalytic or Non-Catalytic Mutations of DNMT3 Enzymes Lead to Overlapping DNAme Alterations

**Figure 3:**
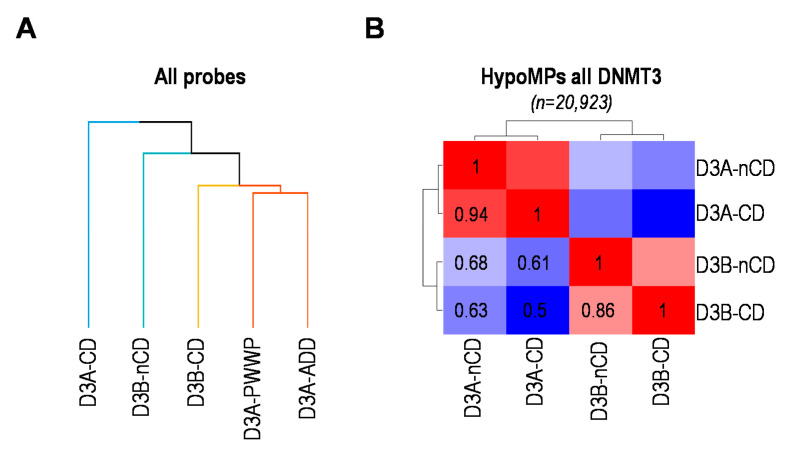
Convergent and distinguishing methylomes in patients with catalytic or non-catalytic mutations in DNMT3 enzymes. (**A**) Clustering of subgroups of patients with catalytic (CD) or non-catalytic (nCD, i.e., PWWP or ADD) mutations in DNMT3A or DNMT3B based on β-values of all the HM450K probes analyzed (*n* = 361,359). Groups are highlighted by different branch colors. (**B**) Pairwise Pearson correlation between patients with catalytic (CD) or non-catalytic (nCD) mutations in DNMT3A or DNMT3B based on the union of all HypoMPs in DNMT3 mutants. The colors of the heat map range from high correlation in red to low correlation in blue. Pairwise indexes are indicated on the heat map.

Hierarchical clustering of all patients in the study, on the basis of all the probes that have passed the quality controls, did not clearly distinguish patients with CD or nCD mutations in DNMT3A or in DNMT3B (Figure 1A). Consistent with the inclusion of patients under the same diagnosis, i.e., TBRS or ICF1, respectively, this suggested that pathogenic variants of these enzymes have redundant consequences for DNAme profiles regardless of their localization with respect to DNMT3 functional domains. However, hierarchical clustering restricted to HypoMPs identified in ICF1 and TBRS patients revealed substantial differences that allowed to discriminate the impact of mutations in DNMT3 CD or nCD domains, with the exception of mutations in PWWP or ADD domains of DNMT3A that could not be discriminated using this criterion (Figure 3A).

Hence, from then on, we grouped these patients into patients with non-catalytic mutations of DNMT3A (D3A-nCD). The correlation plots based on all HypoMPs identified in patients with DNMT3 mutations highlighted that the consequences of CD or nCD mutations in a given DNMT3 were highly correlated (r = 0.94 for DNMT3A-CD vs. nCD; r = 0.86 for DNMT3B-CD vs. nCD) (Figure 3B). In contrast, and consistent with previous findings of distinct (epi)genomic features of HypoMPs in ICF1 and TBRS patients (Figure 2A), these correlation plots highlighted a poor correlation between DNM3A and DNMT3B CD (r = 0.5) or nCD (r = 0.68) mutants (Figure 3B), further emphasizing the non-redundant activities of DNMT3A and DNMT3B at CpG sites.

This remarkable similarity of the DNAme profiles downstream of CD and nCD mutations of DNMT3 enzymes was further illustrated by the number of their shared HypoMPs (Supplementary Figure S9B). Out of the 11,094 and 7686 total HypoMPs, and compared to the 5152 and 2539 HypoMPs unique to DNMT3B nCD or CD mutants, respectively, we found 4887 HypoMPs were shared between these two DNMT3B categories of mutants (~40% of non-redundant HypoMPs in DNMT3B mutants). Despite the fact that nCD mutants of DNMT3B led to a higher degree of hypomethylation compared to CD mutants (11,094 vs. 7686 HypoMPs), this somewhat significant redundancy was also illustrated by the similarities in (epi)genomic features of the HypoMe probes in each category with respect to CpG content, gene and chromatin segmentation features (Supplementary Figure S10A,B). In contrast, we found only poor overlap of DNAme profiles between patients with DNMT3A CD and nCD mutants, with nCD mutants of DNMT3A having a more modest impact on DNAme profiles than DNMT3A CD mutants. Out of the 1353 and 6980 total HypoMPs, and compared to the 465 and 5532 HypoMPs unique to DNMT3A nCD or CD mutants, respectively, we found 518 HypoMPs shared between these two DNMT3A mutant categories (~8% of non-redundant HypoMPs in DNMT3A mutants) (Supplementary Figure S9B). This modest redundancy was also reflected in the (epi)genomic features of the HypoMPs (Supplementary Figure S10A,B). However, the chromatin features of HypoMPs in DNMT3 mutants clearly distinguished DNMT3A from DNMT3B mutations (Supplementary Figure S10B).

In sum, the highly correlated methylome profiles in patients with mutations in CD or nCD domains of DNMT3B strongly suggest that DNMT3B guiding to methylation sites is primarily through interplay between its PWWP domain reading histone H3K36 methylation and calls for further comparison with methylation profiles in Sotos or Sotos-like patients.

### 2.6. NSD1 and DNMT3B Mutations Lead to Overlapping DNAme Alterations at Heterochromatin Domains

**Figure 4:**
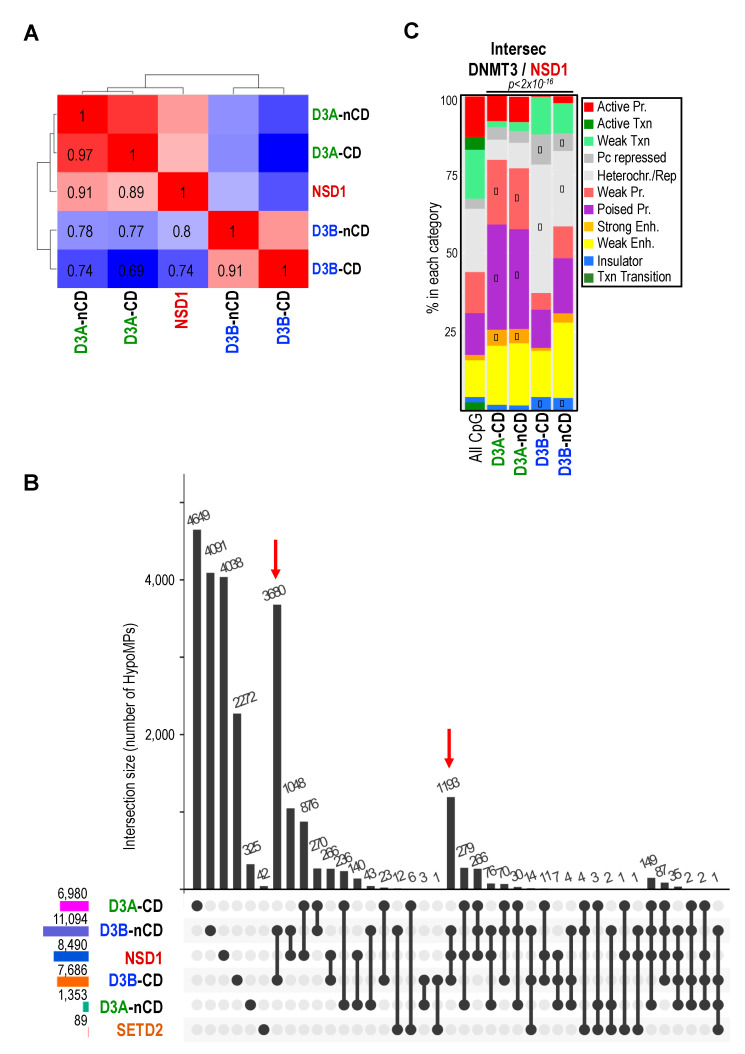
Shared hypomethylated sites between patients with mutations in DNMT3 enzymes and H3K36me2 KMT. (**A**) Pairwise Pearson correlation between patients with mutations in NSD1 or in DNMT3 enzymes based on HypoMPs were identified in NSD1 mutants (*n* = 8490). The colors of the heat map range from high correlation in red to low correlation in blue. The pairwise index is indicated on the heat map. (**B**) Quantitative visualization of HypoMPs overlap between groups of patients was done using UpSet plot (R version). The bottom left horizontal bar graph shows the total HypoMPs identified in patients with mutations in the catalytic (CD) or non-catalytic (nCD) domains of DNMT3A or DNMT3B, and in the H3K36 KMTs NSD1 and SETD2. The circles in each panel’s matrix represent what would be the different Venn diagram sections (unique and overlapping DMPs). Connected dots indicate intersection of HypoMPs between groups. The top bar graph in each panel summarizes the number of HypoMPs for each unique or overlapping combination. (**C**) Epigenomic annotation of the HypoMPs shared between patients with mutations in NSD1 or DNMT3 catalytic (CD) or non-catalytic (nCD) domains as in Figure 2A. *p*-values (p, Chi-square test) assess significant changes in the distribution of HypoMPs within the different categories relative to HM450K array composition. The diamond symbol denotes categories that contribute the most to the *p*-values based on the standardized residuals.

The growing body of evidence, mainly from murine models, of a crosstalk between DNMT3 and H3K36 KMTs to shape DNA profiles motivated the comparison of DNAme alterations in ICF1, TBRS, Sotos and Sotos-like patients. As mentioned above, comparative methylome highlighted the strong correlation of DNAme alterations, resulting from DNMT3 or NSD1 mutations as seen with the highest number of uniquely shared HypoMPs between Sotos and ICF1 (1636 HypoMPs) or Sotos and TBRS (1085 HypoMPs) (Supplementary Figure S9A). We then broke down DNMT3 mutants into CD and nCD mutants. Besides the already mentioned strong correlation between CD and nCD DNMT3B mutants (r > 0.9), we found that the highest correlation was between NSD1 and nCD mutants of DNMT3A (r = 0.91) or DNMT3B (r = 0.8), as well as with DNMT3A CD mutant (r = 0.89) (Figure 4A).

The largest intersected set of shared HypoMPs was between NSD1 and DNMT3B mutants regardless of the position of the mutation (1193 probes) (Figure 4B). Patients with CD mutants of DNMT3A and nCD mutants of DNMT3B shared 876 and 1048 unique HypoMPs, respectively (Figure 4B). The nCD mutants of DNMT3B also shared the highest number of HypoMPs with SETD2, although the very few hypomethylated sites in SETD2 mutants did not allow for further meaningful analysis. Hypomethylated sites common to DNMT3B and NSD1 mutants were mostly intergenic, within CGIs, and corresponded to loci with heterochromatin features in hESCs (Figure 4C and Supplementary Figure S11). In contrast, shared HypoMPs between DNMT3A (both CD and nCD) and NSD1 mutants were more associated with “poised promoter” categories defined in hESCs.

**Figure 5:**
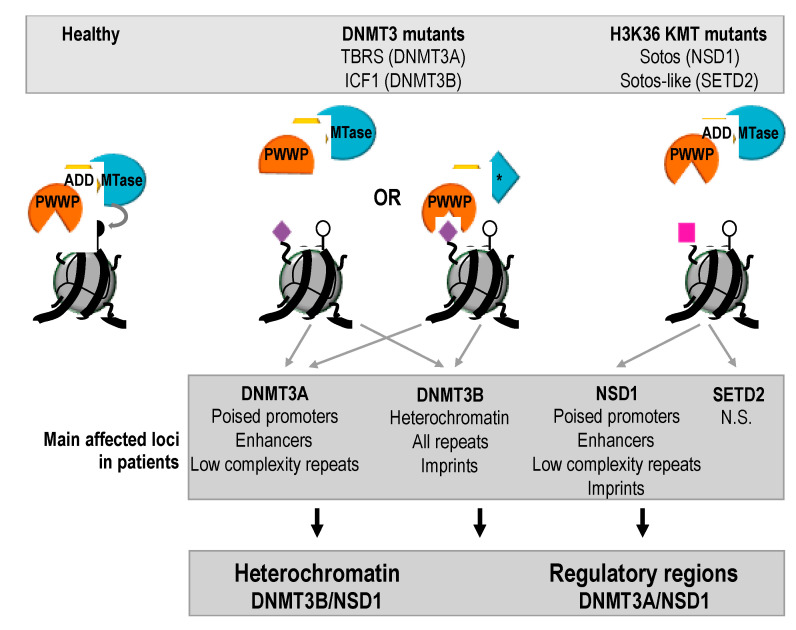
Crosstalk between DNMT3 enzymes and NSD1 seen through comparative analysis of patients methylomes. DNMT3 enzymes are represented in the form of their two regulatory domains PWWP and ADD, and their catalytic domain (MTase). We focus here on histone H3K36me2/me3 (purple diamond) catalyzed by NSD1 or SETD2 KMTs (not represented). DNMT3 mutations in patients can affect the PWWP domain, which can no longer recognize H3K36me2/me3, or the calatytic domain, both leading to reduced DNAme (white lollipop). Likewise, mutations in H3K36me3 KMTs lead to reduced methylation of H3K36 (pink square) and genome-wide DNAme alterations, probably through altered recruitment of DNMT3 enzymes at methylation sites. The main features of affected loci in each class of patients are listed, and intersected in broad classes of chromatin states.

In sum, comparative methylome analysis in patients with mutations in prominent and connected actors of DNAme pathways supported a crosstalk between H3K36me2 and DNAme in humans, probably with distinct genomic preferences with DNMT3A/NSD1 mostly contributing to methylated states of regulatory elements, whereas DNMT3B/NSD1 would be more specifically required for DNAme in portions of the genome with heterochromatin landmarks (Figure 5).

## 3. Discussion

We performed a comparative methylome analysis in patients with rare hereditary diseases caused by LoF mutations in epigenetic regulators with the dual objective to continue efforts in the identification of DNAme signatures shown to improve their diagnostic yield, and to feed our knowledge on the determinants of DNAme at specific locations in the genome and on the interplay between DNA and histone methylation.

### 3.1. DNA Methylation Profiling for Diagnosis and Understanding of Clinical Manifestations

Not surprisingly, DNAme profiling in patients with pathogenic variations in factors of the epigenetic machinery was sufficient to categorize subjects according to their genotype, which reinforced the relevance of using disease-specific DNAme biomarkers for diagnostic purposes when clinical signs are difficult to interpret or genetic origin is unknown or caused by Variants of Unknown Significance (VUS) [30,56,57]. In that respect, the case of ICFX patients is a good example of the benefits brought by methylome profiling in the clinic. Based on methylation biomarkers identified in a mouse model for ICF1 [58] and validated in a cohort of ICF1 patients [43], two ICFX patients without mutation in DNMT3B, ZBTB24, CDCA7 or HELLS were classified as ICF1-like based on typical DNAme signatures of DNMT3B LoF, which included hypomethylation of germline genes promoters and pericentromeric satellite repeats but excluded hypomethylation at alpha-satellite repeats [43]. This signature also served to diagnose a child born to a consanguineous family, before traditional cytogenetics analysis [59] and targeted sequencing had been performed. Yet, because multi-locus alteration of DNAme landscapes is a hallmark of many pathological situations [60,61], the use of such a limited number of DNA biomarkers is probably not always selective enough to establish a reliable diagnosis. In that respect, the recent development of genome-wide DNAme profiling for the screening and classification of rare developmental disorders with overlapping clinical features and unclear genetic origin provided a real breakthrough. Here, based on thousands of DMPs, we could unambiguously classify ICFX patients as ICF1 and orientate etiological research towards DNMT3B-related pathways.

We also provided the first methylome profiling of Sotos-like patients, so-called because they present with an incomplete phenotype of Sotos caused by mutations in the H3K36 trimethylase SETD2 instead of mutations in the H3K36me2 dimethylase NSD1 [36,37]. DNAme profiles were clearly distinct between Sotos and Sotos-like patients, as well as between Sotos-like and other diseases. This was consistent with these diseases being distinct, although extensive overlap of clinical signs between SETD2-associated phenotypes and other overgrowth syndromes related to NSD1, EZH2 or DNMT3A in Sotos, Weaver and Tatton-Brown-Rahman syndromes, respectively, has been reported [36,37]. Hence, methylome profiling provides an interesting tool to orientate mutation screening in overgrowth syndromes when ambiguous facial dysmorphism and ID can confound accurate diagnosis.

However, these findings contradicted the general trend towards highly similar DNAme profiles that was reported in different subtypes of Kabuki syndrome or other chromatinopathies [32]. In fact, the little DNAme alterations in Kabuki syndrome rather suggested that DNAme defects in such a disease might be too low or limited to too few genomic regions to distinguish among subtypes, which also barely distinguished patients from healthy subjects. Likewise, we previously reported the striking distinct methylome profiles in subgroups of ICF patients under the same diagnosis, which discriminated ICF1 patients with mutations in DNMT3B from ICF2-4 patients with mutations in non-DNMT factors [39]. Here, we reported that hypomethylation of satellite DNA repeats, as well as hypomethylation within genomic regions with heterochromatin features, is a unique feature of the ICF syndrome that does not seem to be shared by other rare genetic disorders of the epigenetic machinery. Strikingly, DNAme profiling segregated patients according to the methylation status of alpha-satellite repeats, although how this relates to the distinguishing DNAme alterations on the rest of the genome [39] or variable clinical manifestations [62] remains poorly understood. Recently, a direct link between DNA hypomethylation of satellite repeats, increased levels of their derived transcripts and an interferon-based innate immune response has been established in a Zebrafish model for the ICF2 syndrome [63]. This aberrant activation of the innate immune system was suggested to explain autoimmunity that affects some of the ICF patients [64]. In addition, recent studies have highlighted a role for ZBTB24, CDCA7 and HELLS in Non-homologous end joining (NHEJ), a pathway involved in DNA repair but also in immunoglobulin class-switch recombination, linking LoF of ICF factors to immunodeficiency that affects most of ICF patients [41,65].

Patients with overgrowth syndromes, although they could be discriminated based on their distinguishing methylation profiles, exhibited widely overlapping DNAme alterations, including at developmentally regulated clusters of genes that encode key regulators of embryonic development and patterning. We found that alteration of DNAme patterns at HOX genes was a common feature of all patients with overgrowth syndromes, including Weaver syndrome, although it seemed limited to *HOXA5* in this particular case (not shown and [66]). Recent data showed that aberrant DNAme at these developmentally regulated genes was associated with dwarfism in mouse and human with DNMT3A mutations [67,68]. Of note, DNAme profiles at these gene clusters were also affected in ICF1-X patients, although to a lesser extent. Thus, alterations to DNAme profiles in developmentally regulated genes are likely to have adverse consequences for human growth.

Because developmental anomalies and ID are the main clinical features shared by patients with LoF of epigenetic factors [69], we also analyzed the CpG methylation status of parentally imprinted gene loci, many of which are important for the regulation of fetal or placental growth and development, while others play key roles in neurological pathways and behavior [70,71]. The allele-specific expression of imprinted genes is orchestrated by parental allele-specific DNAme imprints that are acquired in germ cells and maintained in all somatic lineages throughout development [72]. Here, we found that the most striking DNAme loss at both ICRs and non-ICR regions was found in ICF1 patients with DNMT3B mutations. This observation was quite unexpected in light of experimental evidence from mouse models where Dnmt3a is the de novo DNMT that plays a predominant role in the establishment of parental imprints, in collaboration with its co-factor Dnmt3l [73]. Whether DNMT3B contributes to establishment or maintenance of DNAme imprints in human remains to be formally tested, but our data suggest that DNMT3A may be dispensable in humans. Other mechanisms for the recruitment of DNMT3A/DNMT3L on ICRs include removal of, or failure to establish, H3K4me, or read-through mechanisms [38]. The significant CpG hypomethylation at ICRs in Sotos patients with mutations in a H3K36 KMT, but not in Kabuki patients with mutations in a H3K4 KMT, suggests that acquisition of DNAme imprints could be associated with active transcription in humans. Along these lines, NSD1 LoF in Sotos patients has been recently correlated with hypomethylation of two imprinted DMRs [74]. In mice, NSD1 and SETD2 have been shown to establish H3K36me2 or H3K36me3 in male germ cells or oocytes and to be required for de novo DNAme at paternal or maternal imprints, respectively [75]. This contrasts with DNAme data from human patients, where we found that parental imprints are not affected by SETD2 mutations, whereas both maternal and paternal imprints are affected by NSD1 mutations. In a more clinical perspective, the discovery of NSD1 mutations in Beckwith-Wiedemann syndrome (BWS) [76], an imprinted and congenital overgrowth syndrome, could indicate a causal link between NSD1 LoF, alterations to parental imprints and overgrowth phenotype. However, this link is not that straightforward since parental imprints were more dramatically affected in ICF1 patients who exhibit growth delay, whereas they were mostly not affected in the other overgrowth TBRS and Sotos-like syndromes. It is interesting to note that arrays of tandem repeat motifs are frequently found in or close to germline DMRs and were suggested to have a role in imprint acquisition [38,77]. Hence, whether the rather selective hypomethylation of both parental imprints and repetitive elements in ICF patients are linked and related to reduced occupancy of DNMT3B at these loci is an interesting point that deserves attention.

In sum, blood-based methylation biomarkers have been recognized to be representative of DNAme alterations in numerous tissues of the affected individuals and to discriminate patients according to their genotype. However, studies from human diseases make it difficult to fully understand the relationship between DNAme alterations and early onset emergence of clinical signs, such as ID or growth defects, which will benefit from the development of new and sophisticated disease modeling tools such as brain organoids to dissect molecular and cellular mechanisms [78].

### 3.2. DNA Methylation Profiling in Patients to Predict Determinants of DNAme in Humans

One of the major issues in the field of DNAme is to identify the molecular actors and mechanisms that specifically guide DNMTs at certain genomic locations. Since DNAme is relatively stable once established, widespread DNAme alterations in patients with monogenic germline mutations are likely to reflect primary errors in the establishment of DNAme profiles, which are then perpetuated throughout life (reviewed in [30]). Since these alterations can result from pathogenic variations in a wide-range of epigenetic factors, whether they act directly or indirectly on DNAme, it is, therefore, tempting to infer functional links between these factors, or at least question findings in human patients in light of mechanisms established in animal models.

A striking example was the discovery of mutations in factors of unknown function but devoid of DNMT activity, namely ZBTB24, CDCA7 and HELLS [79,80], as genetic causes of the ICF syndrome. The distinct methylomes among ICF patients contradicted the hypothesis that ZBTB24, CDCA7 and HELLS would serve as platforms for the recruitment of DNMT3B early during development, when most of the DNAme patterns are established (this study and [39]). Similarly, the lack of DNAme overlap between ICF2-4 and TBRS patients suggested that, if ZBTB24, CDCA7 and HELLS have a direct link with the DNAme machinery, it is probably not through DNMT3A, although one should be careful when comparing the impacts of recessive germline and dominant mutations could be misleading. It is possible that ZBTB24/CDCA7/HELLS form a hub that cooperates with the maintenance DNAme enzyme DNMT1, as supported by recent studies [41,81]. These data would also explain the widespread hypomethylation that characterizes ICF2–4 patients. In light of the essential role of the chromatin remodeler HELLS in shaping DNAme patterns in mice [82], especially at DNA repeats, it is tempting to speculate that its nucleosome remodeling activity is essential for DNMT1targeting to methylation sites. Yet, the loading of HELLS onto chromatin was proposed to rely on CDCA7, whose expression is itself dependent on the integrity of ZBTB24 [39,83,84]. Hence, we believe that our vision of the links between ZBTB24, CDCA7, HELLS and DNMTs or DNAme at specific genomic loci remains fragmentary.

The strong correlation of the methylation patterns we observed in the patients with DNMT3 mutations in its catalytic or non-catalytic domains was also very striking and suggested that DNMT3 guiding to methylation sites may primarily rely on its ability to recognize histone modifications through its PWWP domain. The first case report of ICF1 patients carrying homozygous mutations in the PWWP domain of DNMT3B was instrumental in our understanding of the molecular mechanisms that guide DNMT3 activity at specific places on the genome [85]. These results were supported by elegant genetic and biochemical studies that also demonstrated how CpG methylation at specific genomic sites was dictated by the ability of the PWWP domain of DNMT3 to read changes in histone marks H3K36me2/me3 [10,54,86,87,88].

In addition to the PWWP domain, our data also illustrate the contribution of the ADD domain for the targeting of DNMT3A activity onto the genome. This ADD domain, also present with the PWWP domain in the N-terminal part of DNMT3, can recognize histone H3 tails unmethylated at lysine K4. This binding of the ADD domain to the chromatin allows conformational changes of DNMT3 and releases the autoinhibition of DNMT3 exerted by the ADD on the catalytic domain [16,89,90,91].

Although being an attractive mechanism, the contribution of the PWWP and ADD domains in the targeting of DNMT3 activity on the genome is probably not sufficient to explain how it shapes methylation landscapes. An important point to understand how DNAme is established and by which factors, is to also answer the question of “when” the considered factors begin to be expressed during development. As illustrated by the distinct DNAme profiles in TBRS and ICF1 patients, and in light of genome-wide methylome studies performed in Dnmt3 knock-out mouse models [5,73,92,93], the shaping of DNA methylomes by DNMT3 enzymes appears to rely on multiple factors including the timing of expression of DNMTs during development [94], their respective interactions with transcription factors [9] as well as the genomic context of the CpGs as shown by recent studies [53].

Based on comparative analyses of the methylation profiles in rare genetic diseases, it is clear that patients with ICF1, TBRS and Sotos share a large part of their respective methylation signatures. This result may reflect the existence of the afore mentioned crosstalk between H3K36 and CpG methylation, which involve the PWWP domain of DNMT3 enzymes. Several recent studies have reported that the distribution of DNMT3 activity at euchromatin regions of the genome is orchestrated by the presence of H3K36me2 and H3K36me3 catalyzed by the NSD1 and SETD2 enzymes, respectively [10,54,55,87]. In addition, experimental evidence highlighted the preference of DNMT3B for H3K36me3 mark at active gene bodies, whereas DNMT3A preferred intergenic regions enriched in H3K36me2 [10,54]. Although globally consistent, at least with regard to the high similarity between methylomes of TBRS and Sotos patients, the mechanistic models of DNMT3 recruitment at H3K36me2/me3 enriched-chromatin seem to be partially conserved between mouse and human. In fact, the methylation defects downstream of DNMT3B or SETD2 mutations were quite distinct, probably primarily due to the very modest impact of SETD2 LoF on CpG methylation in Sotos-like patients. Yet, this was unexpected in light of data in mice showing that H3K36me3 mediates the targeting of Dnmt3b activity [10], but consistent with a recent study showing that the invalidation of the *SETD2* gene in human HEK293T cells did not affect overall CpG methylation [55]. Hence, and although SETD2 was thought to be a non-redundant H3K36 trimethylase, it is possible that its LoF is compensated by another enzyme, such as SETD5, which haploinsufficiency affected H3K36me3 levels in knockout mice [95] and is associated with severe ID in human [96]. It is also possible that the persistence of H3K36me2 marks in SETD2 mutants [97,98] could compensate the loss of H3K36me3 for guiding DNMT3 activity, provided that H3K36me2 is a substrate for trimethylation by SETD2.

In contrast to SETD2 LoF, we observed that NSD1 LoF in Sotos patients caused significant CpG hypomethylation in the human genome, to the same extent as DNMT3 LoF. This result strongly supports a conserved role for NSD1 activity to shape DNA methylome in mouse and human [54]. Interestingly, we found that among all the patients included in this study, patients with NSD1 or DNMT3B mutations, including catalytic and non-catalytic DNMT3B mutations, shared the highest HypoMPs suggestive of an under-appreciated cooperative pathway between NSD1 and DNMT3B activities for CpG methylation in human, most likely through a crosstalk between H3K36me2 and the PWWP domain of DNMT3B, which has been poorly documented so far. However, we cannot rule out the possibility that NSD1 LoF also has an impact on levels of H3K36me3 mark if H3K36me2 serves as a substrate for H3K36 trimethylation [14].

The CpG hypomethylation common to Sotos and ICF1 patients strikingly overlapped genomic regions with heterochromatin features. This observation is quite novel and interesting since the crosstalk between H3K36me2/me3 and CpG methylation has only been documented for euchromatin regions so far [10,54]. An attractive hypothesis would be that the NSD1 methyltransferase activity is not restricted to the H3K36 substrate. This hypothesis is supported by results showing that lymphocytes from Sotos patients exhibit, in addition to defects in H3K36me2 and H3K36me3, a loss of H4K20me3 marks [99], the latter being a mark enriched within heterochromatin. Interestingly, it has been documented that PWWP domains would also have the ability to read the H4K20me3 mark [100]. As a consequence, it is possible that the CpG hypomethylation within heterochromatin regions common to Sotos and ICF1 patients may in fact reflect a crosstalk between H4K20me3 and the CpG methylation activity of DNMT3B. This may also explain the reduced DNAme at parental imprints in Sotos patients, which are also more modest that in ICF1 patients, that we reported here, since the methylated allele of imprinted genes is consistently associated with repressive histone marks, including H4K20me3 [38].

### 3.3. Conclusions

Beyond the clinical perspectives for diagnosis, our study also illustrates how the comparative DNAme profiling of patients with rare monogenic disorders can contribute to the field of research on the determinants of DNAme in humans. Our results clearly underline the important role played by NSD1 in shaping human DNA methylomes in cooperation with the de novo DNMT3 enzymes and their properties as readers of histone methylation. Consistent with non-redundant functions for DNMT3s, and despite their highly similar structural domains, we can assume that they also have relatively distinct functional interactions with NSD1 along which NSD1 would cooperate with DNMT3A for DNAme at developmentally regulated genes or with DNMT3B at imprinted and heterochromatin loci. Yet, our understanding of the dynamics of DNAme and the expression of its players during human development, as well as our knowledge of the molecular guides for DNMT3 enzymes as methylation sites, are still in their infancy. However, (epi)genomic studies in genetic disorders of the epigenetic machinery will continue to make a significant contribution to this widely studied field.

## 4. Materials and Methods

### 4.1. Sample Preparation for Genome-Wide DNA Methylation Analysis and Quality Controls

Peripheral whole-blood from ICF, Luscan-Lumish (Sotos-like) and TBRS patients (Supplementary Figure S2A) served to extract genomic DNA using Gentra Puregene DNA Extraction kit (Qiagen, Courtaboeuf, France) following manufacturers recommendations. One microgram of DNA was deaminated with the EZ-96 DNA Methylation Kit (Zymo Research, Irvive, CA, USA), a simplified procedure that streamlines bisulfite treatment of DNA. Hybridization on the Illumina^®^ Infinium Methylation EPIC Array, which interrogates 860,000 CpG sites across the human genome (compared to 450,000 CpG sites on HK450K) and fist-steps quality controls were outsourced to Diagenode following their workflow (https://www.diagenode.com/en/p/infinium-methylation-epic-array-service; accessed on 1 Ferbuary 2020 ). For all the samples, the qualitative metrics were validated for further analysis. The proportion of failed probes was <0.1 and the genome-wide DNA methylation density showed a bimodal distribution.

### 4.2. Datasets of DNA Methylation Arrays Used

Whole blood-based DNAme data, generated using the Illumina Infinium methylation 450K (HM450K) bead chip Illumina, San Diego, CA, USA), from ICF, Sotos, Kabuki, TBRS and healthy subjects were retrieved from the Gene Expression Omnibus (GEO http://www.ncbi.nlm.nih.gov/geo/; accessed on 1 March 2021) database with the following accession numbers: GSE95040, GSE74432, GSE97362, GSE128801.

### 4.3. Processing of DNA Methylation Array Data

The Bioconductor R package (https://www.bioconductor.org/; accessed on 1 April 2020) Chip Analysis Methylation Pipeline ChAMP [101] was used to analyze DNAme raw data. HM450K and EPIC arrays raw Intensity Data files (.idat) were loaded using the *champ.load* function. We filtered out probes with a detection *p*-value > 0.01, a bead count <3 in 5% of samples, non-CpG content, SNP-related [102], multi-hits [103] and probes located on sex chromosomes. Due to the different design of probes (Infinium type I and II) or the Bead Chip array used (HM450K or EPIC), we performed a multi-samples quantile normalization to make the distribution of probe intensities comparable before differential analysis using the ChAMP package. In short, data were normalized using beta-mixture quantile normalization (BMIQ) with the function champ.norm(norm = ’’BMIQ’’), and then corrected for batch effects using champ.runCombat function. Because samples were assayed using two different platforms (HM450k and EPIC), only the probes shared between the two types of arrays were considered before normalization and correction of batch effects. The methylation levels for each probe were measured as a beta value (β). Beta-values for each probe were computed using the classical formula: β = max(M,0)/[max(M,0) + max(U,0) + 100], where, for a given probe, M and U correspond to the methylated and the unmethylated signals intensities, respectively, and ranged from 0 (no methylation) to 1 (full methylation).

For the comparative analysis between two genotypes, we computed the mean of the probe β-value for each genotype and discarded probes with a detection *p*-value > 0.01. For autosomal probes analysis, male and female samples were normalized together excluding probes located on sexual chromosomes. We considered a probe as differentially methylated (DMP) between two genotypes when the mean β-value difference was ≥0.2 (interpreted as 20% methylation difference).

### 4.4. Visualization of DNA Methylation Data

Hierarchical clustering representations, Heat maps, Volcano plots, Bar plots, correlation calculations and intersection visualization were generated using functions in R software environment for statistical computing. Sample clustering and heat map visualizations were performed using the dendextend, factoextra and ComplexHeatmap R packages (https://www.bioconductor.org/; accessed on 1 April 2020). For hierarchical clustering, the hclust and heatmap.2 R functions were used with “euclidean” and “complete” method parameters, respectively, for distance and agglomeration. Volcano plots were generated using the EnhancedVolcano R package. Basic and stacked bar plots were build using the ggplot2 R package. The Upset R package was used to visualize intersection of sets of DMPs.

### 4.5. Statistical Analysis

The identification of Differentially methylated probes (DMPs) was performed using the champ. DMP function (adjPVal = 0.05), which implements the limma package to calculate the *p*-value for differential methylation using a linear model [104]. *p*-values were adjusted (adjPVal) with a Benjamini-Hochberg correction.

The Gene ontology enrichment analysis was performed using the clusterProfiler R package. The significant GO categories were identified with Benjamini-Hochberg adjusted *p*-value (*p*.adjust) ≤ 0.05.

For the annotation of probes relative to the various categories defined in terms of CpG density, genomic and epigenomic contexts, the proportion of probes assigned to each category was calculated, and their distribution compared through a Chi-square goodness of fit test (chisq.test R function) using R programming environment.

The significance of differences in DNAme levels (β-values) between healthy subjects (CTL) and patients (ICF1-X, ICF234, Sotos, TBRS and Sotos-like) at ICR, non-ICR and classes of DNA repeat was estimated using a Wilcoxon rank-sum test (wilcox.test R function) using R programming environment.

### 4.6. Annotation of DMPs and Databases Used

CpG annotation was performed using the Infinium Methylation450K manifest file, according to their location relative to CpG islands (UCSC_CpG_Islands_Name). Gene annotation of the HM450K array probes was performed using GENCODE release 36 gene annotation originally created on the GRCh38 reference chromosomes, mapped to the GRCh37 primary assembly (https://www.gencodegenes.org/human/ accessed on 1 April 2020). Functional annotation of CpG sites was obtained using Intersect intervals tools of bedtools (2.29.0) to compare DMPs to the following publicly available databases: Repeat Masker (http://www.repeatmasker.org/; accessed on 1 June 2020), Imprinted loci [50,51] and ENCODE ChromHMM states (Chromatin State Segmentation by HMM from ENCODE/Broad [105].

## Data Availability

The data from this study have been submitted to the NCBI Gene Expression Omnibus (GEO; http://www.ncbi.nlm.nih.gov/geo/; accessed on 1 March 2021) under accession number GSE167230.

## Supplementary Materials

The following items are available online at https://www.mdpi.com/article/10.3390/ijms22073735/s1: Figure S1: Patients included in the study and their clustering according to their DNA methylation profiles. Figure S2: New patients enrolled in the study. Figure S3: Analysis of the genomic context of reduced DNAme in patients. Figure S4: Gene Ontology of genes linked to DMPs in a given disease. Figure S5: DNA methylation status of DNA repeats in each patient. Figure S6: DNA methylation status of satellite DNA repeats in each patient. Figure S7: DNA methylation landscapes at developmentally regulated clusters of genes in disease groups. Figure S8: DNA methylation landscapes at HOX genes clusters in disease groups. Figure S9: Hypomethylated sites specific or shared between patient groups. Figure S10: (Epi)genomic annotation of HypoMPs in patients with mutations in DNMT3 catalytic or non-catalytic domains. Figure S11: Genomic annotation of HypoMPs shared between patients with NSD1 or DNMT3 mutations. Tables S1 to S6: Annotation of DMPs in diseases. Tables S7 to S10: Gene ontology enrichment analysis of DMPs in diseases.
