# Supplementary material for: Interplay between Histone and DNA Methylation Seen through Comparative Methylomes in Rare Mendelian Disorders
Source: Int J Mol Sci. 2021 Apr 3;22(7):3735. doi: 10.3390/ijms22073735 (PMC8038329; doi:10.3390/ijms22073735)
Supplement: Supplementary file 1 [file ijms-22-03735-s001.zip › Velasco_Supplementary Figures S1 to S11_R1.pdf]

A

Syndrome (# patients)	OMIM	Genes	Chr.	Functions	Source
**ICF1 (8)**	242860	DNMT3B	20q11.21	DNMT-CD	GSE95040
**ICF1 (1)**	242860	DNMT3B	20q11.21	DNMT-nCD	**This study**
**ICF2 (4)**	614069	ZBTB24	6q21	TF	GSE95040
**ICF3 (2)**	616910	CDCA7	2q31.1	TF	GSE95040
**ICF4 (1)**	616911	HELLS	10q23.33	Chromatin remodeller	GSE95040
**ICFX (2)**	ND	ND	ND	ND	**This study**
**Kabuki (11)**	147920	KMT2D	12q13.12	KMT (H3K4me1/me2)	GSE97362
*** Luscan-Lumish (2)**	616831	SETD2	3p21.31	KMT (H3K36me3)	**This study**
**Sotos (38)**	117550	NSD1	5q35.3	KMT (H3K36me2)	GSE74432
**TBRS (4)**	615879	DNMT3A	2p23.3	DNMT-CD	GSE128801
**TBRS (2)**	615879	DNMT3A	2p23.3	DNMT-nCD	**This study**

B

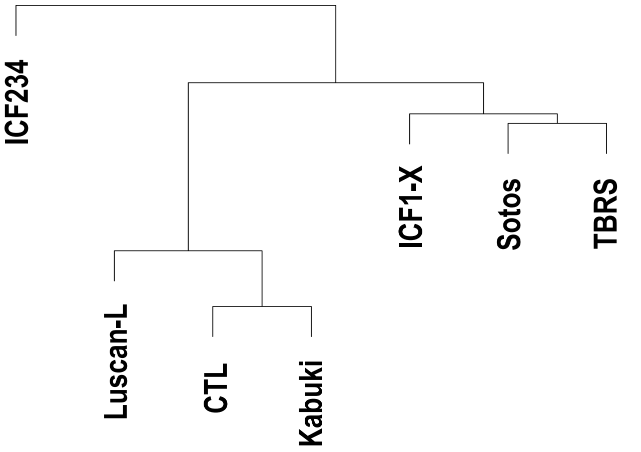

Supplementary Figure S1. Patients included in the study and their clustering according to their DNA methylation profiles. (**A**) Patients included in the study and the corresponding available array-based methylome datasets. Lines in grey indicate newly established methylomes. #: number of patients; chr: chromosome number; DNMT: DNA methyltransferase; KMT: Histone lysine methylase; CD: catalytic domain; nCD: non-catalytic domain; TF: transcription factor; source: GEO accession numbers of publicly available DNA methylation array-based methylomes; ND: not determined; * also known as Sotos-like. (**B**) Clustering of patients based on β-values of all the arrays probes that passed quality controls (n=361,359)

A

Syndrome (sexe, age)	Patients	Genes	Chr.	Mutations	Status	Clinical signs
**ICF1** (M,11 y.)	P282	DNMT3B	20q11.21	p.S270P g.808T>C	Ho	Shirohzu et al., 2002
**ICFX** (F, 3 y.)	pN	ND	ND	ND	Cons.	Carla et al., 2004
**ICFX** (F,4 y.)	pBMH	DNMT3B	20q11.21	p.S655L g.1964C>T	Het	Sterlin et al., 2015
**Luscan-Lumish** (M,25 y.)	Sotos-like P1815	SETD2	3p21.31	p.L1815W g.544T>G	Het	Overgrowth and ID
**Luscan-Lumish** (M,9 y.)	Sotos-like P2452	SETD2	3p21.31	p.S2452L g.7335C>T	Het	Overgrowth and ID
**TBRS** (M,14 yrs)	P307	DNMT3A	2p23.3	p.P307L g.25470554G>A	Het	Overgrowth and ID
**TBRS** (M,13 yrs)	P595	DNMT3A	2p23.3	p.L595R g.25467091A>C	Het	Overgrowth and ID

B

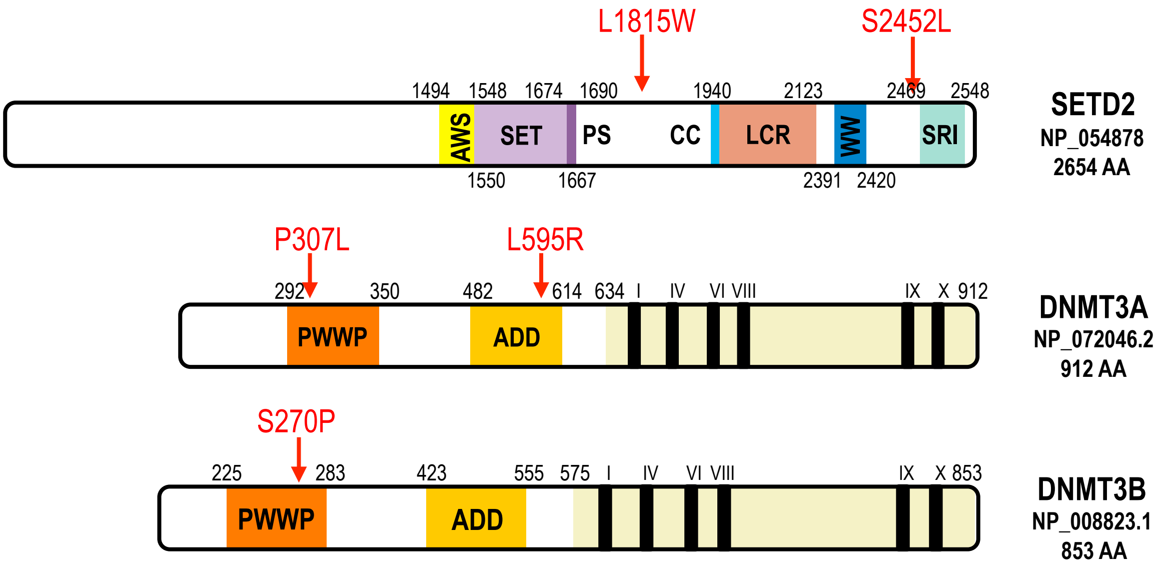

Supplementary Figure S2. New patients enrolled in the study. (**A**) Mutations and main clinical signs of newly enrolled patients for which DNA profiling had not been performed before. y.: years old; Chr: chromosome number; ND: not determined; Ho: homozygous; Het: Heterozygous; Cons: from a consanguineous family; ID: Intellectual Deficiency. (**B**) Location of the mutations are indicated by red arrows on the schematic representation of SETD2, DNMT3A and DNMT3B proteins. The corresponding amino-acid changes are indicated on top. Protein domains are indicated by color blocks and marked by residue positions. AWS, Associated With SET domain; SET, [Su(var)3-9, Enhancer-of-zeste and Trithorax] domain; PS, post-SET domain; CC, coiled-coil domain; LCR, low-charge region; WW, domain with two invariant tryptophan (W) residues; SRI, Set2 Rbp1 Interacting; PWWP, proline-tryptophan-tryptophan-proline domain; ADD, ATRX-DNMT3-DNMT3L-type zinc finger domain. The C-terminal part of DNMT3 proteins contains the catalytic domain (yellow box), characterized by conserved motifs indicated by Roman numerals.

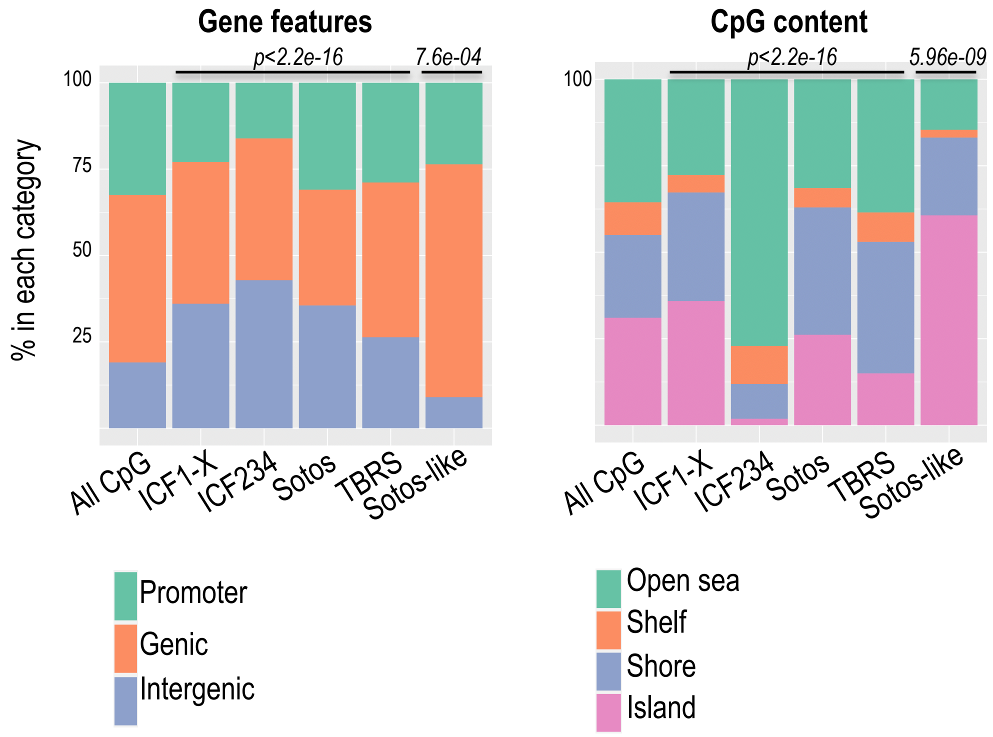

Supplementary Figure S3. Analysis of the genomic context of reduced DNAme in patients. Distribution of HypoMPs relative to gene features (on the left) and CpG content (on the right). The distribution of the total number of probes analyzed in these categories is also shown (All CpG = 361,359 probes). P-values (p, Chi-square test) assessing significant changes in the distribution of category relative to All CpG are indicated.

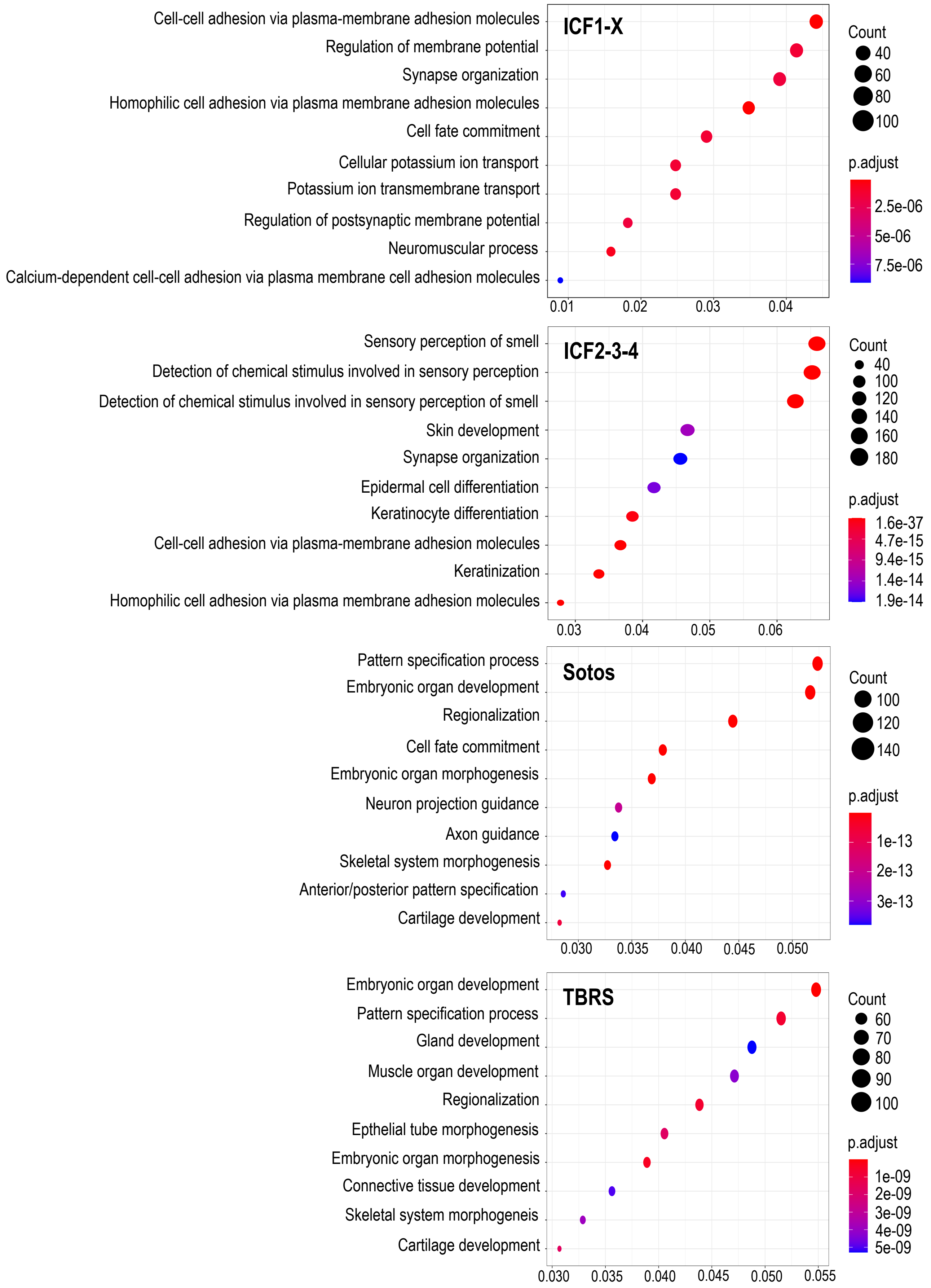

Supplementary Figure S4. Gene Ontology of genes linked to DMPs in a given disease. Dot plots represent enrichment in Biological Processes. GeneRatio on the x axis corresponds to the number of genes linked to DMPs divided by the number of genes annotated in the corresponding process. The size and colors of the dots indicate gene counts and the adjusted p-value for the GO pathway listed on the y axis, respectively. Only the top 10 GO terms with the highest degree of enrichment are represented.

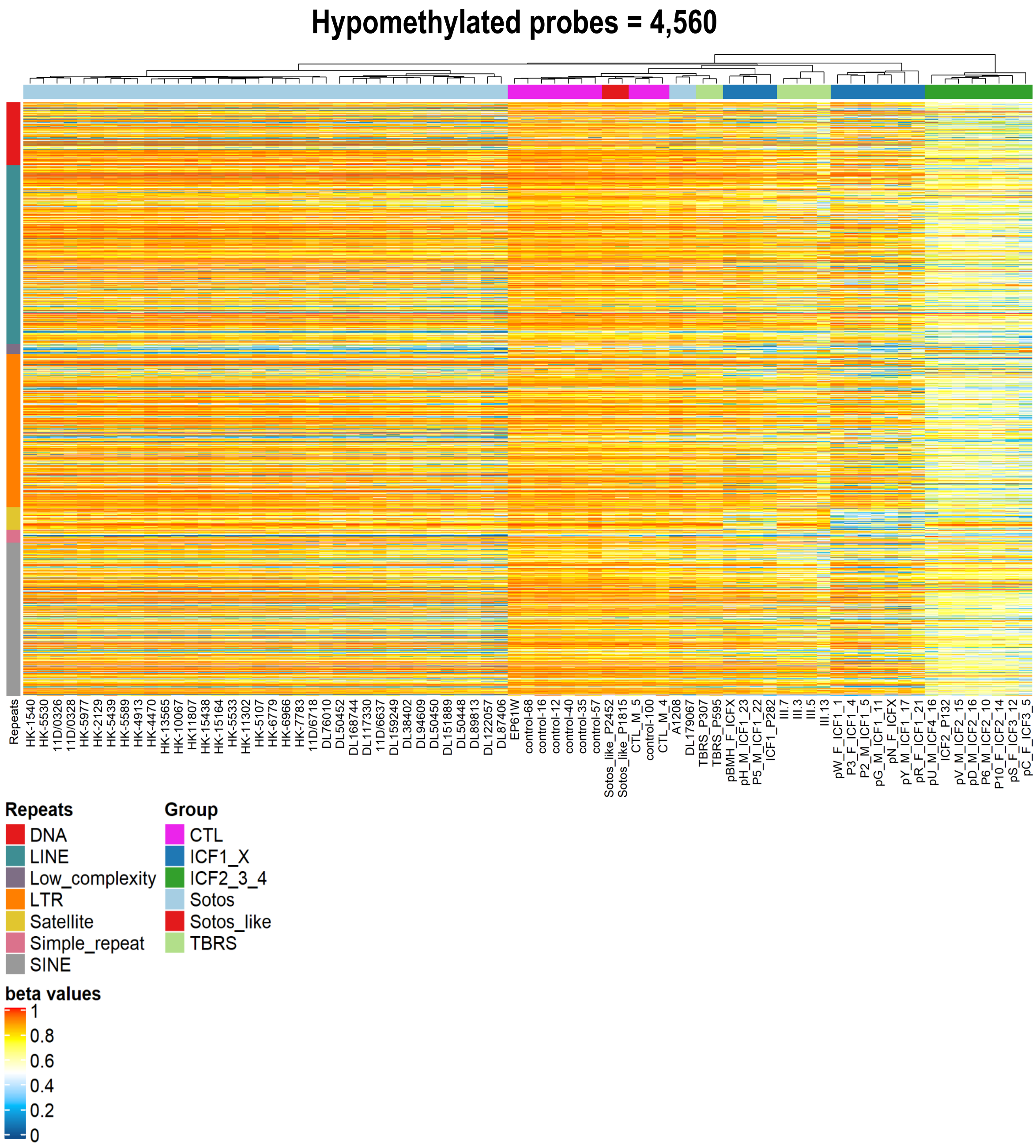

Supplemental Figure S5. DNA methylation status of DNA repeats in patients. Clustering analysis with heat map of β-values of the 4,560 probes of the HM450K designed in different classes of DNA repeats. High methylation levels are shown in red and low methylation levels in blue, according to the scale bar of beta-values at the bottom of the figure. Each column represents an individual and each row represents a probe. The colored sidebar on the left represents each class of DNA repeat, and the top colored bar represents the different disease groups. The color codes are indicated below the heat map.

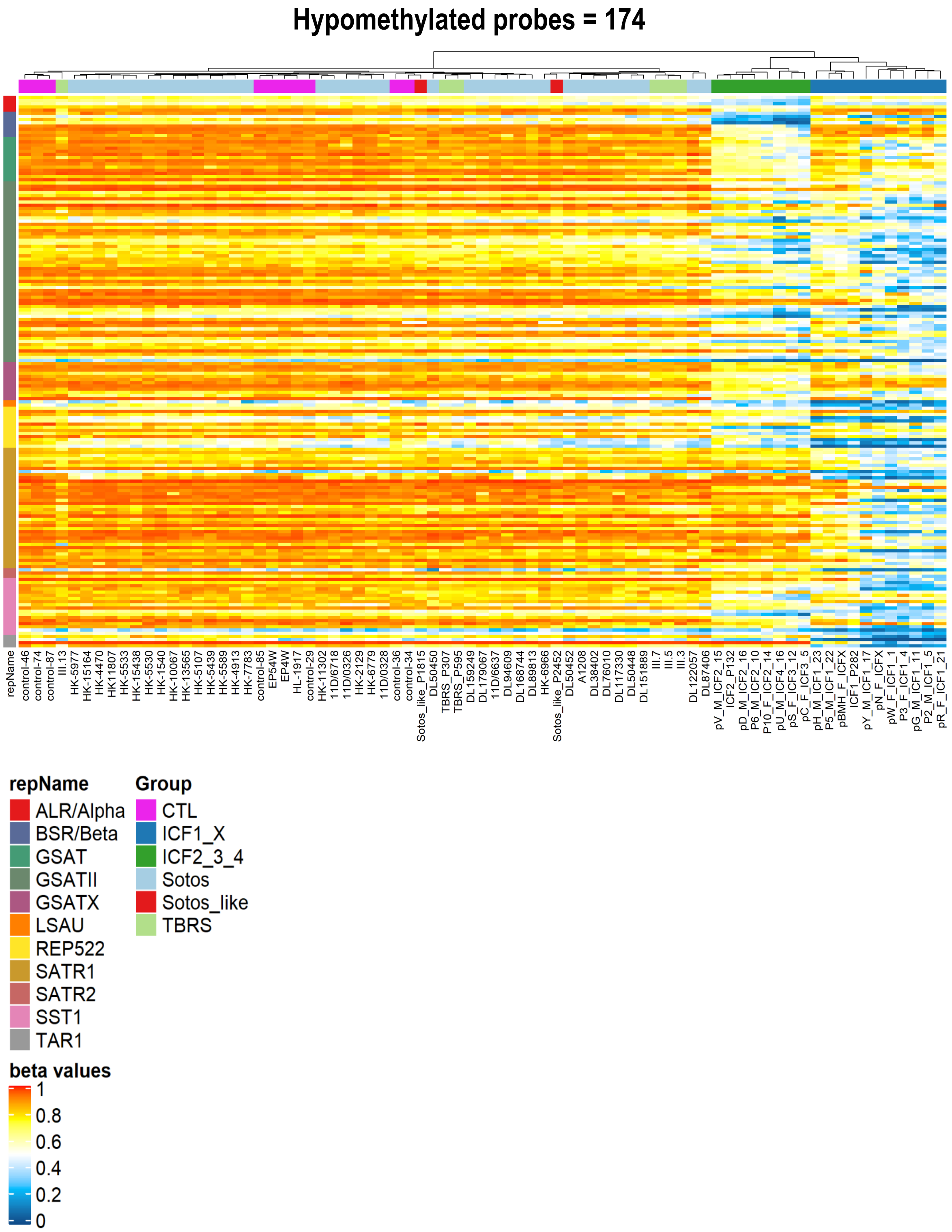

Supplemental Figure S6. DNA methylation status of satellite DNA repeats in patients. Clustering analysis with heat map of β-values of the 174 probes of the HM450K designed in satellite DNA repeats. High methylation levels are shown in red and low methylation levels in blue, according to the scale bar of beta-values at the bottom of the figure. Each column represents a subject and each row represents a probe. The colored sidebar on the left represents each class of satellite DNA repeat, and the top colored bar represents the different groups of diseases. The color codes are indicated below the heat map.

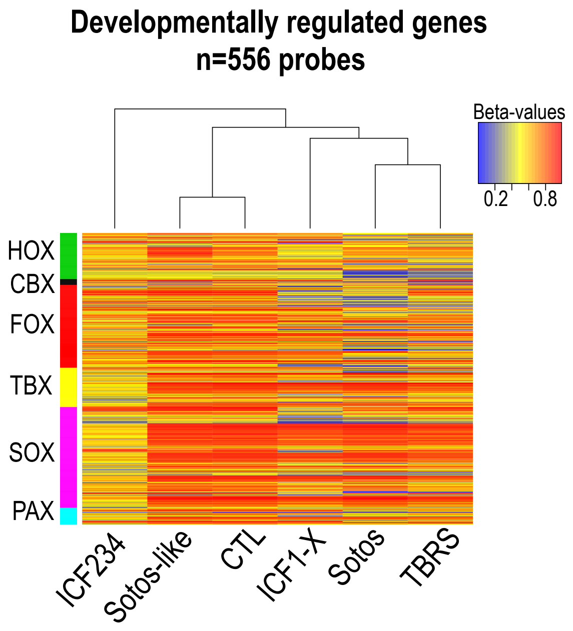

Supplementary Figure S7. DNA methylation landscapes at developmentally regulated clusters of genes in disease groups. Clustering analysis with heat map of β-values of DMPs probes designed in developmentally-regulated genes encoding HOX, CBX, FOX, TBX, SOX and PAX transcriptional regulators. High methylation levels are shown in red and low methylation levels in blue, according to the scale bar of beta-values at the top of the figures. Each column represents a group of subjects and each row represents a probe. The colored sidebar on the left represents each cluster of developmentally-regulated genes .

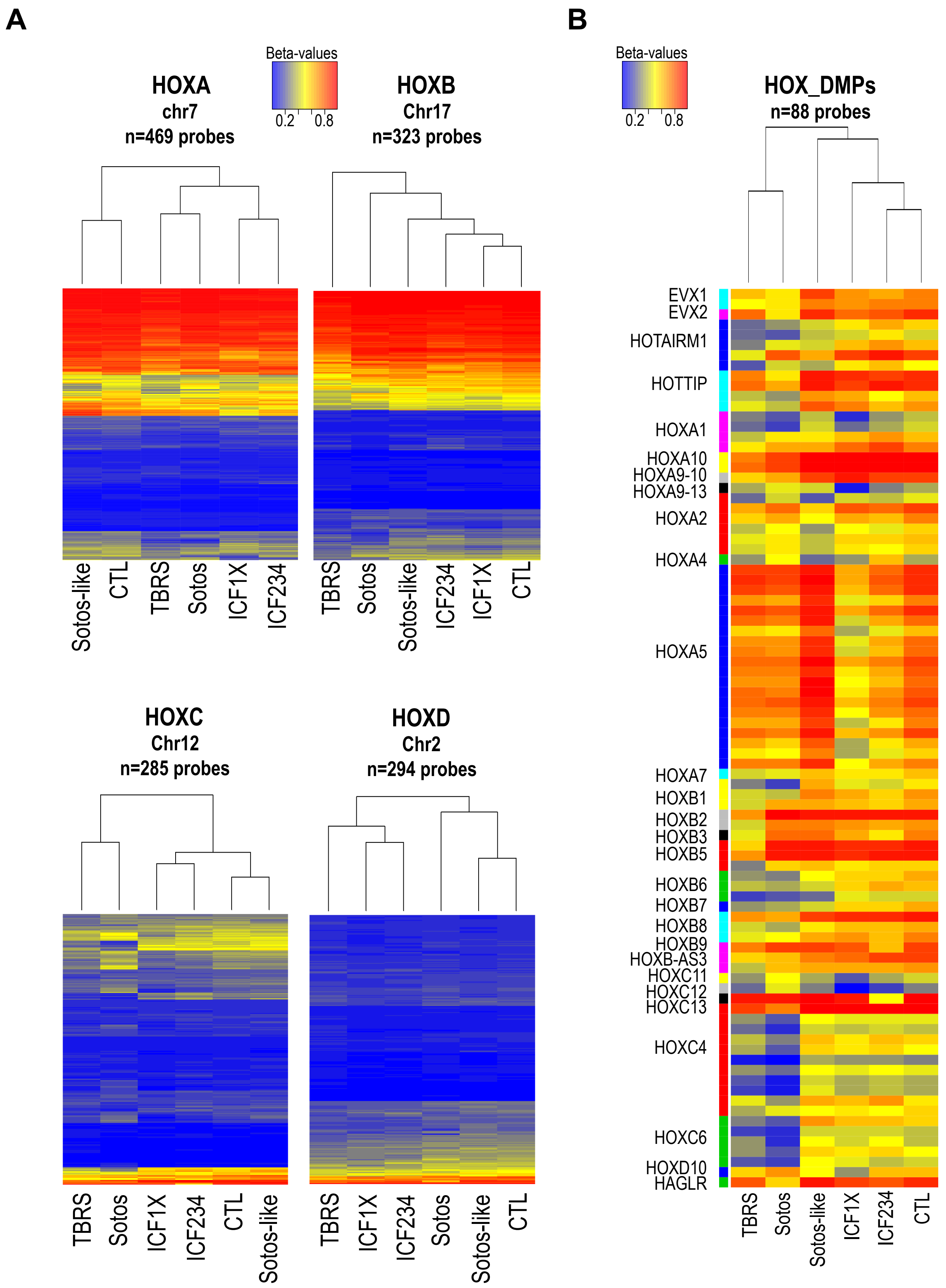

Supplementary Figure S8. DNA methylation landscapes at HOX genes clusters in disease groups. (**A**) Unsupervised hierarchical clustering of disease groups on the basis of β-values of HM450K probes designed in HOXA (n=469), HOXB (n=323), HOXC (n=285) and HOXD (n=294) gene clusters. (**B**) Unsupervised hierarchical clustering of disease groups on the basis of β-values of DMPs across the HOX genes (n=88). High methylation levels are shown in red and low methylation levels in blue, according to the scale bar of beta-values at the top of the figures. Each column represents a group of subjects and each row represents a probe.

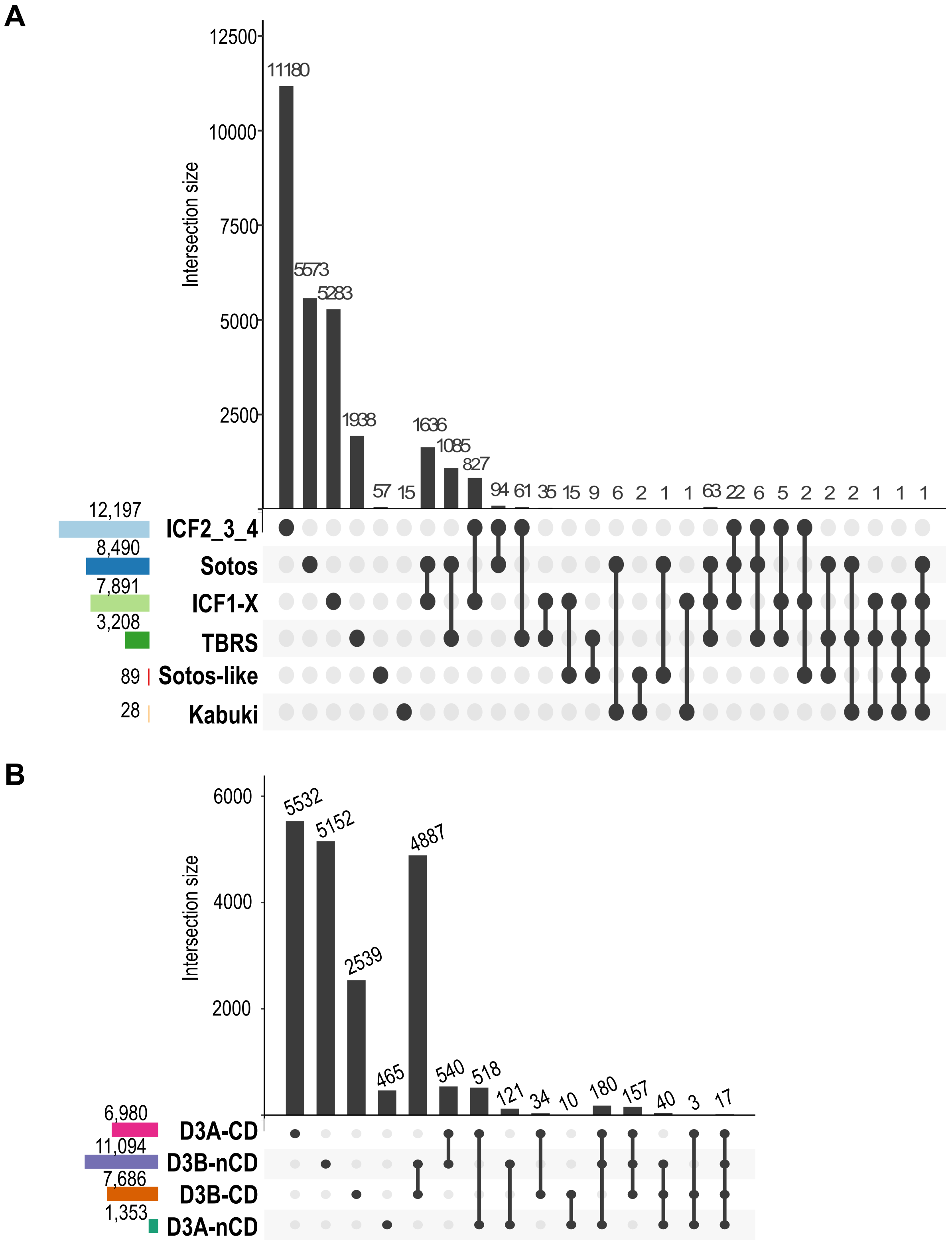

Supplementary Figure S9. Hypomethylated sites specific or shared between patient groups. (**A**) Quantitative visualization of HypoMPs overlap between patient groups, using UpSet plots (R version). The bottom left horizontal bar graph shows the total HypoMPs identified in ICF2-3-4, Sotos, ICF1-X, TBRS, Sotos-like and Kabuki patients. The circles in each panel's matrix represent what would be the different Venn diagram sections (unique and overlapping DMPs). Connected dots indicate intersection of HypoMPs between groups. The top bar graph in each panel summarizes the number of HypoMPs for each unique or overlapping combination. (**B**) UpSetR plot comparing groups of patients with catalytic (CD) and non-catalytic (nCD) mutations in DNMT3A or DNMT3B.

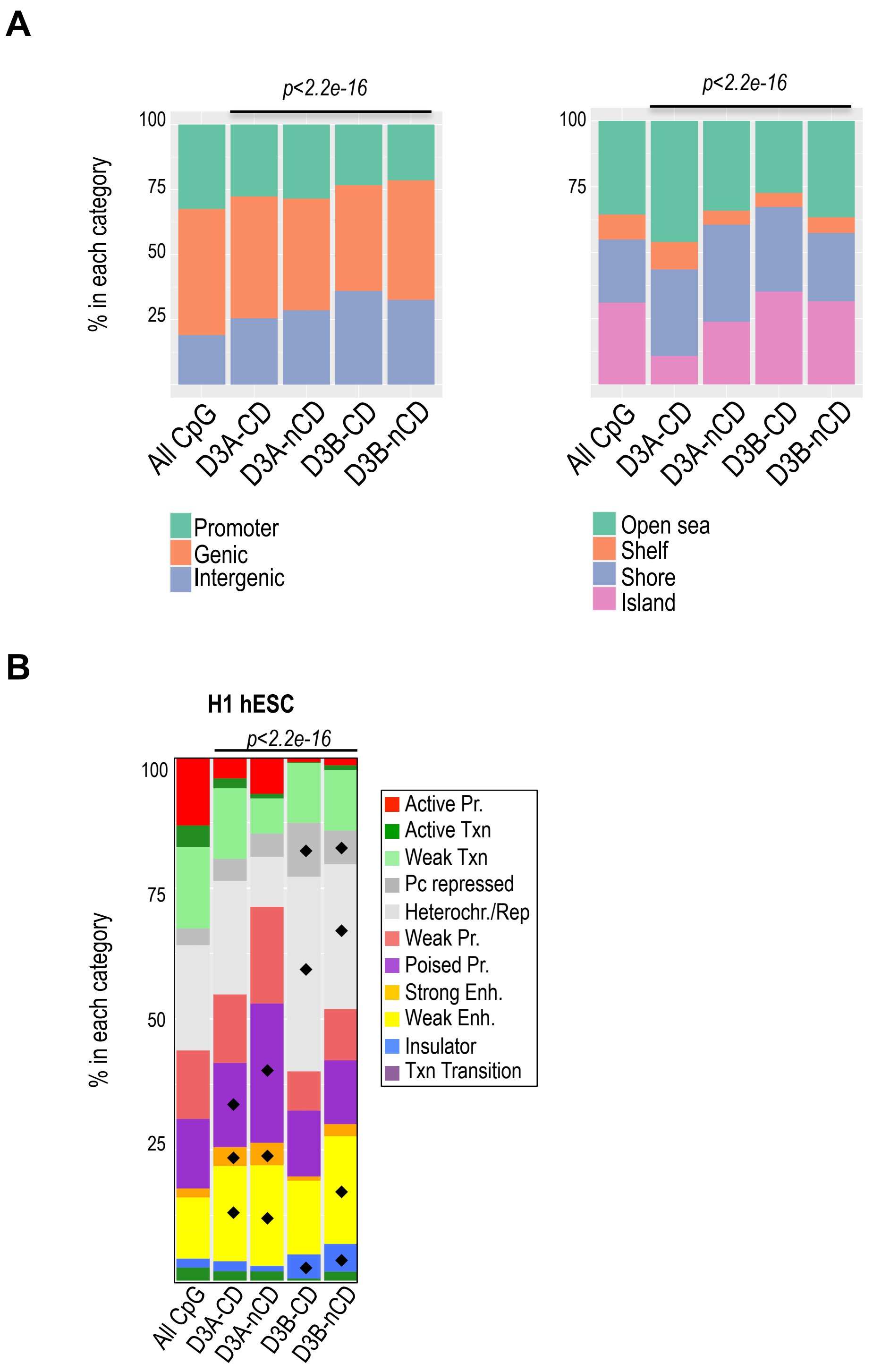

Supplementary Figure S10. (Epi)genomic annotation of HypoMPs in patients with mutations in DNMT3 catalytic or non-catalytic domains. (**A**) Distribution of HypoMPs downstream of catalytic (CD) and non-catalytic (nCD) DNMT3 mutants, relative to gene features (on the left) and CpG content (on the right). The distribution of the total number of probes (361,359) analyzed in these categories is shown (All CpG line). (**B**) Annotation of HypoMPs for catalytic (CD) and non-catalytic (nCD) DNMT3 mutants according to the 11 chromatin states indicated on the right (ChromHMM from ENCODE/Broad) established in H1 hESCs. P-values (p, Chi-square test) assessing significant changes in the distribution of HypoMPs within the different categories relative to HM450K array composition. The diamond symbol denotes categories that contribute the most to the p-values based on the standardized residuals.

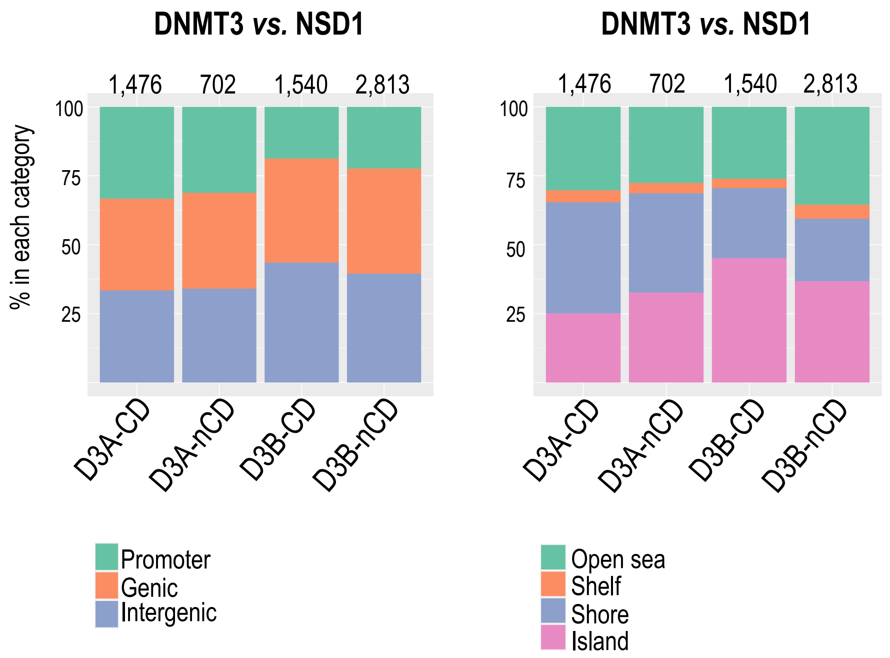

Supplementary Figure S11. Genomic annotation of HypoMPs shared between patients with NSD1 or DNMT3 mutations. Distribution of shared HypoMPs relative to gene features (on the left) and CpG content (on the right). The number of probes in each intersection is indicated on the top.
